# Situating commercial determinants of health in their historical context: a qualitative study of sugar-sweetened beverages in Jamaica

**DOI:** 10.1186/s12992-023-00962-5

**Published:** 2023-09-12

**Authors:** Olivia Barnett-Naghshineh, Sheray Warmington, Henrice Altink, Ishtar Govia, Karyn Morrissey, Matthew J. Smith, Ruth H. Thurstan, Nigel Unwin, Cornelia Guell

**Affiliations:** 1 European Centre for Environment and Human Health, University of Exeter, Penryn, TR10 9FE UK; 2 Epidemiology Research Unit, Caribbean Institute for Health Research (CAIHR), The University of the West Indies, Mona, Kingston, 7 Jamaica; 3 Department of History and Interdisciplinary Global Development Centre, University of York, Heslington, York, YO10 5DD UK; 4 Institute for Global Health, University College London, London, WC1N 1EH UK; 5 Climate and Energy Policy, Department of Technology, Management and Economics, Technical University Denmark, Kgs. Lyngby, 2800 Denmark; 6 Department of History, University College London, London, WC1E 6BT UK; 7 Centre for Ecology and Conservation, University of Exeter, Penryn, TR10 9FE UK; 8 Medical Research Council Epidemiology Unit, University of Cambridge, Cambridge, CB2 0SL UK

**Keywords:** Commercial Determinants of Health, Sugar Sweetened Beverages, Jamaica, Nutritional Colonialism, History, Qualitative Research

## Abstract

**Background:**

Non-communicable diseases (NCDs) are the leading cause of mortality across the Caribbean and similar regions. Structural determinants include a marked increase in the dependency on food imports, and the proliferation of processed foods, including sugar-sweetened beverages (SSBs). We focused on Jamaica as a case study and the health challenge of SSBs, and situated contemporary actions, experiences and policies within their historical context to investigate underlying drivers of commercial determinants of health and attempts to counter them. We asked: how can a historical perspective of the drivers of high level SSB consumption in Jamaica contribute to an enhanced understanding of the context of public health policies aimed at reducing their intake?

**Methods:**

An ethnographic approach with remote data collection included online semi-structured interviews and workshops with 22 local experts and practitioners of health, agriculture and nutrition in Jamaica and attending relevant regional public webinars on SSBs and NCD action in the Caribbean. Our analysis was situated within a review of historical studies of Caribbean food economies with focus on the twentieth century. Jamaican and UK-based researchers collected and ethnographically analysed the data, and discussed findings with the wider transdisciplinary team.

**Results:**

We emphasise three key areas in which historical events have shaped contextual factors of SSB consumption. *Trade* privileged sugar as a cash crop over food production during Jamaica’s long colonial history, and trade deregulation since the 1980s through structural adjustment opened markets to transnational companies. These changes increased Jamaican receptiveness to the mass *advertisement and marketing* of these companies, whilst long-standing power imbalances hampered taxation and *regulation* in contemporary public health actions. Civil society efforts were important for promoting structural changes to curb overconsumption of SSBs and decentring such entrenched power relations.

**Conclusion:**

The contemporary challenge of SSBs in Jamaica is a poignant case study of commercial determinants of health and the important context of global market-driven economies and the involvement of private sector interests in public health policies and governance. Historically contextualising these determinants is paramount to making sense of the sugar ecology in Jamaica today and can help elucidate entrenched power dynamics and their key actors.

## Background

Non-communicable diseases (NCDs) have become a significant global health burden [1], and are now the leading cause of mortality in low- and middle-income countries (LMICs) [2]. Small Island Developing States, including those in the Caribbean, have some of the highest rates of NCDs globally [1]. In Jamaica, for example, NCDs accounted for 80% of all deaths in 2018, and around 1 in 6 adults will die from an NCD before their 70th birthday [1]. Poor nutrition, especially through the consumption of foods high in sugar, fat and salt is a major contributor to the risk of developing many NCDs, including type 2 diabetes, cardiovascular diseases and several cancers [3].

Public education programmes about healthy diets have been shown to have some limited impact on lowering the consumption of foods that are high in sugar, fat and salt, and health literacy more generally is important [4]. However, it is increasingly recognised by public health scholars that this alone cannot solve the problem. The structure of the economy, policy environment and food systems (including environmental factors and industry) all contribute to the health burdens caused by NCDs, globally and particularly in LMICs like Jamaica [5]. A principal issue is the increased availability of sugar-sweetened beverages (SSBs) in the Caribbean, Latin America and the Pacific resulting from trade liberalisation that started in the 1980s on the insistence of international financial institutions, such as the World Bank and International Monetary Fund (IMF). Trade liberalisation has been a major driver of a “nutrition transition” whereby ultra-processed, pre-cooked foods high in salt, sugar and fat (including trans-fats) have become more readily available, accessible and affordable in these regions [6–9].

The transition is particularly alarming in the consumption rates of SSBs. Jamaica has one of the highest per capita consumption rates of SSBs worldwide [10]. Sales of commercial SSBs increased by more than 40% between 2014 and 2016 [10, 11]. SSBs make up the largest contribution of sugar intake and are particularly harmful. They increase the risk of type 2 diabetes contributing significantly to higher overweight and obesity levels and through independent negative metabolic effects. On average, a person who is overweight and consumes one SSB per day has around 18% greater risk of developing type 2 diabetes than an overweight person who does not [12, 13].

In this paper, we argue that the long-entrenched dynamics of *nutritional colonialism* are an important context to understanding SSB consumption in Jamaica. In particular, this concept helps to explore commercial drivers for the wide consumption of sweetened drinks and barriers to counter these. According to Diana Burnett [14], *nutritional colonialism* refers to a contemporary and continuing colonial dynamic which sees people in post-colonial contexts adopt new consumption patterns, and subsequently suffer health consequences, while transnational food corperations increase their profits. This concept points to the historical context of commercial drivers (including marketing, distribution, production, profit) that encourage higher consumption. In our investigation, we aimed to answer the following research question: how can a historical perspective of the drivers of high level SSB consumption in Jamaica contribute to an enhanced understanding of the contemporary context of public health policies and actions aimed at reducing their intake?

In asking this question, we aim to contribute to the field of *commercial determinants of health* that brings into focus not only the “unhealthy commodities contributing to ill-health” – SSBs in this study – but also the “business, market and political practices that are harmful to health and used to sell these commodities and secure a favourable policy environment” such as advertisement and lobbying, and increasingly also point to “global drivers of ill-health, such as market-driven economies and globalisation” (15, p.1). This reflects a recent broad definition of commercial determinants of health to encompass “the systems, practices, and pathways through which commercial actors drive health and equity.” (16, p.1195) In this paper, we suggest including historical perspectives to this approach to understand how these global but also local dynamics have been established and ingrained. To do so, we argue that entrenched dynamics of *nutritional colonialism* facilitate the domination of SSB industries.

Using critical ethnographic inquiry, we qualitatively explored the perspectives of key stakeholders and experts on leading structural challenges [17], and contextualised these in a historically grounded way. We argue that the historical and contemporary conditions of globalisation affecting Jamaica are important to understand the barriers to NCD policy action, despite the ongoing dedication and energy of regional public health actors [18, 19].

## Methods

### Research design and participants

We chose an ethnographic research design to understand the role of sugar and SSBs in the NCD crisis in Jamaica through multiple expert perspectives. Due to restrictions of the Covid-19 pandemic, this was a fully online study and we adapted principles from digital ethnography to inform design, logistical and ethical considerations [20]. Twenty-two online interviews were conducted between March and August 2021, through a collaboration between an anthropologist based in the UK (OBN) and a political scientist based in Jamaica (SW). In addition, we held a bespoke workshop with local researchers and attended relevant public webinars on SSBs and NCD action in the Caribbean to understand current academic, policy and practice debates in the region.

For our participants, we recruited a purposive sample of public health experts, food growers, sugarcane farmers, sweetened beverage company owners (including manufacturers of SSBs), civil society actors and researchers who have worked on policies, and in practice, in the areas of nutrition, food and agriculture, advertising, marketing and governance. These included a senior clinical public health researcher who has experience in policy and practice with expertise in NCDs and drivers of food systems, a technical officer of a health nongovernmental organisation (NGO) with many years of experience in public advocacy and community engagement, and a public historian with over 35 years of experience working in the sugarcane industry as a technician. The commercial sector was represented by a senior member of management in a major drinks distribution company, and a restaurant manager and farm manager of a globally renowned organic food outlet in Jamaica. Finally, we received advice in several information gathering conversations with long-standing experts in NCD third sector responses in the region. For the sake of confidentiality and anonymity we have kept the identities for each quote general.

Interviewees were initially recruited through our established stakeholder networks for their considerable knowledge of local and regional research, practice or policy in the relevant areas and sectors identified above. We snowballed from there to actively seek out perspectives from the private sector, civil society, research and agriculture through contacting agencies and organisations (e.g. the All-Island Jamaica Cane Association) in relation to our initial research questions. Our online research approach enabled a broad range of connection and collaboration with interviewees based in Jamaica, but also in Barbados, Trinidad and Tobago and those from the region who were based in the US and UK when we spoke with them [21].

The research design and research question built on previous projects, including a transdisciplinary partnership-building award that connected public health researchers with historians. In this previous pilot study (conducted in 2019) we analysed fast-food advertisement in *The Gleaner* newspaper archives (one of the major daily newspapers and the oldest paper in Jamaica) to understand changes over time in Jamaica’s foodscape and explored in what way a transdisciplinary research agenda between historians and health researchers can inform contemporary challenges [22].

### Data collection

Interview guides were developed by the team as well as approved by the University of Exeter and University of West Indies ethics committees. The interviews were semi-structured and allowed for flexibility in dialogue. The interviews were transcribed by a third party and checked over by the researchers. Our two primary researchers (OBN and SW) conducted the semi-structured interviews using Zoom or WhatsApp, as preferred by the interviewee. The researchers also reviewed digitally available documents from the Jamaican government archive and the *Gleaner* archive of policy briefs, ministerial speeches, newspaper articles, and followed contemporary public discourses through social media and online newspapers. They attended online seminars and meetings with specific relevance to Jamaica, health and NCDs, between policymakers and academics as participants and presented the research ideas at the local University.

### Data analysis

The ethnographic analysis was developed through themes emerging from interviews and in iterative dialogue with critical social theory, critical colonial and post-colonial histories of Jamaica and the Caribbean and conducted between the two lead researchers (OBN and SW) and the principal investigator (CG). Critical input was provided from co-authors with specialisms in Caribbean history (HA and MJS), public health (IG, NU), ecology (RHT) and economics (KM), as well as from selected interviewees and partners of the project, with whom we shared our emerging analysis.

## Findings

### Trade

*At the end of the day, what we’re producing as a nation is not even enough to really support our people, so we’re importing onions, we’re importing potatoes, we’re importing rice, which is a huge part of the Jamaican diet […], and we’ve stopped growing rice on the island in any large scale. [...] So our food systems have been totally... they’ve been hijacked. In a way we’re still enslaved, and we don’t even recognise it.*

-*Organic farmer in North-East Jamaica*

As we will show in this section, there are aspects to the current dynamics of the food system in Jamaica today that have their roots in recent histories (Structural Adjustment Programmes and their trade liberalisation) and longer histories (plantation economies).

#### The long history of plantation economies

The contemporary dynamic of significant amounts of food being imported into, and exported from, Jamaica, has a long history, with roots in the sugarcane economy. Although this dynamic has become normal for many countries since neo-liberal globalisation emerged, in Jamaica, the historical context of this early form of globalisation is relevant to the question of how SSB consumption is shaped by commercial determinants and the challenges of shifting this dynamic [23, 24].

During the sixteenth century, European colonisation was brutally established in the Caribbean. This included the decimation of indigenous populations of Caribs and Taino-Awaraks [25] through disease and plunder as their land was co-opted. In the 17th century, Jamaica, along with many other islands of the Caribbean, fertile and tropical lands were claimed by European planters (English, Spanish, French, Danish, Dutch) principally for sugarcane production which thrived in the tropical climate. Sugar required massive capital, land, and labour. After uneven attempts to establish a white indentured labour force in the sugar zones, colonisers resorted to the enslavement of African people for free labour [25]. From the seventeenth century onwards, sugar production in the Caribbean became synonymous with racialised slavery.

In his theory of plantation economies, George Beckford outlines how the Caribbean islands were formed through plantations which depended on geographical dynamics and led to specific social formations [26, 27]. The flatter and more accessible landscapes of islands such as Barbados and Jamaica allowed for deep agricultural intrusion, and the establishment of large-scale industrial agriculture [28, 29]. Pantin, a Caribbean dependency theorist, argues that with the era of enslavement also began a dependency on imported foods which continued beyond the end of slavery [30]. After abolition, people worked in the cane fields for low incomes as a peasantry and were encouraged to consume imported goods by the colonial regime [30]. He states that this “reduced effective demand for locally produced goods and services, and thus the dynamic potential of the residentiary sector” [30, p.19]. For a similar argument, see Higman’s historical account of different foodways and food cultures that developed in this era through the encouragement and planting of particular crops, and led to persistent cultural practices [31]. Alongside such imported goods, enslaved people working on the plantations survived by using their own provision grounds, cultivating “provision foods” such as yams, plantain and other high energy foods. The sugar they were forced to grow and harvest also became an important and instant source of calorific intake. Hence, some of our interviewees suggested that a “sweet tooth” has long existed in Jamaica, since sugarcane has long been a vital source of energy. Thus, as Mintz shows, the politics of sugar and its consumption has always been embroiled with inequities and power [23].

The Caribbean sugar business had radical consequences both for the landscape of Jamaica but also for the food cultures and economies that developed. Sugar was central to the Jamaican diet and environment during and after slavery. Jamaica exported unrefined molasses from their sugar plantations, which was refined elsewhere and then imported back to the island as refined sugar [24]. Brown sugar was refined domestically, while white granulated sugar was refined in countries which could invest in their processing infrastructure. Some sugarcane farmers we spoke with regretted what they perceived as a lack of investment in this level of refinement as they felt Jamaica was left producing a raw product rather than commodities of higher value. Some brown sugar is still produced locally, and molasses are used for alcoholic beverages. Yet, today Jamaica has become primarily an importer of refined sugar and domestic sugar production has been flagging for decades, especially since the government sold off its industry to foreign interests from 2009 onwards [32]. Commercial sweetened beverages made domestically contain imported refined sugar. Some of the sugarcane farmers and extension workers we interviewed acknowledged feeling the impossibility of competing with producers elsewhere (such as Colombia) who refine large quantities of their product. The historical dynamics of sugarcane production shape the current landscape in Jamaica [24], as one of our interviewees described:

*This is a crop that is 500 years within the Caribbean, and that particular crop has infrastructure throughout Jamaica, in every village; every town in Jamaica is designed around sugarcane.*

-*Public historian of the sugar industry*

#### The recent history of trade liberalisation

While these long-term historical dynamics are an important backdrop to contemporary experiences of sugar consumption, the more recent history from Jamaica’s independence from British rule in 1962 point to more immediate commercial drivers. In particular, Jamaica’s experience with Structural Adjustment Programmes, which began in the late 1970s as Jamaica signed a two-year standby agreement with the IMF and was accelerated in the 1990s, had a radical impact on Jamaica’s food system. Structural Adjustment Programmes are a set of conditionalities and economic measures tied to loans by the international financial organisations such as the IMF and typically include privatisation, deregulation and trade liberalisation [33]. In the 1980s, tariffs on food imports were removed making it harder for local farmers to compete with imported crops such as onions, and products such as dairy or milk powder. Jamaica’s markets were opened and soon highly processed food products from the US and globally flooded Jamaica’s markets [34].

The trade liberalisation encouraged during the 1980s and 1990s as a way of boosting economic growth and development in LMICs has had drastic impacts on food systems in Jamaica [35–37] and made Jamaicans vulnerable to the profit-seeking intentions of transnational food and agricultural trade interests. For instance, sugar as an ingredient, and as a finished product in sweetened beverages made elsewhere, began entering Jamaica in large quantities. Analysing SSB-specific trade flows in 44 LMICs, research by Mendez Lopez and colleagues [35] found that for each 10% increase in imports there was an associated rise of 0.36 L in sweetened beverages sales per capita. In other words, when trade policies enable lower trade tariffs, SSB imports can be expected to increase, with a consequence of rising obesity and NCDs associated with increased consumption of SSBs. This has led to cynicism amongst some of the academics and experts that we interviewed or observed in webinars, who felt there is less discretion after trade liberalisation about the types of foods, and their nutritional qualities, that are brought into the country. We were also told directly by a member of the SSB industry that Jamaica is still seen as a “growth market” for sweetened beverages (despite the known health crisis caused by sweetened beverages). An interviewee poignantly described the market opportunity for transnational companies to develop their business in Jamaica.

*We [Jamaicans] are one of the largest consumers of sweetened beverages in the Caribbean; of many islands in the Caribbean, we’re up there. So, we are a market that the food industry would want. and if you look at some of the big manufacturers, many of them have manufacturing plants here. And we have often been seen in the Caribbean as somewhat of a dumping ground for things that may not necessarily pass the standards in other countries, but it’s easy to have it come here.*

-*Nutrition researcher*

### Advertising and marketing

*One of the other big drivers of obesity here is the presence of big food and big soda and marketing... [Transnational fast-food company] is on every corner, you know, and they use regular marketing plus the social media marketing plus everything else. And so, the dietary patterns have changed very dramatically in Jamaica over the past 30, 40 years*

-*Nutrition researcher*

#### Proliferation of fast-food outlets and their mass advertisement

Our earlier study of newspaper advertising in Jamaica since 1945 showed a marked increase from the mid-70s for foreign foods that fit the “Standard American Diet”; burgers, pizzas and fried chicken [22]. US-based fast-food franchises became a common feature in Jamaica, and they especially increased in the 1980s and 1990s. At the same time, transnational (and local) fast food brands and their drinks worked their way into everyday life – seen on billboards, in newspapers, on radio and television. As Burnett [14, p.641] states, transnational fast-food advertising “promote[s] participation in the normativity of Euro-American foods and patterns” at the expense of local, seasonal, and nutritious eating. Jamaicans that choose healthy diets as part of their lifestyles may do so for health reasons but also due to their religion such as Rastafarianism or the increasingly popular vegan movement. However, the more affordable, accessible and desired diet has become high in sugar and fat content. Increasing mass advertisements for SSBs and fast foods on physical billboards, newspapers and digital social media add significantly to the urban and rural landscapes of Jamaica’s sugar economy and environment – including the sugarcane fields [38].

Mass advertisement of unhealthy products is a core commercial determinant of health, and even more so when it explicitly enters the public health sphere [39]. In Jamaica, a recent example is the sponsorship of the government’s covid-19 vaccination programme by private fast-food and sweetened beverage companies. In one instance, a major transnational fast-food company advertised widely on social media to sponsor a covid-vaccination site in Jamaica’s capital Kingston using a poster on Twitter. The poster suggested that anyone who would attend for their vaccine would also get a free meal deal or mobile phone credit. Here we see how the covid-19 pandemic opened up space for food corporations to position themselves as providing a public service, and normalise the unhealthy diets these companies promote in times of need while creating advertisement space in the same instance. This highlights the lack of fiscal space countries such as Jamaica have since Structural Adjustment Programmes, leaving essential services to be provided by private funders. As one civil society health advocate explained:

*They are coming out under the guise of, ‘okay, all these people are there waiting for several hours for the vaccine. Let us be really good citizens and let us come and give them all our Pepsi and our Coke’.*

-*Civil society campaigner*

The partnering of the government with fast-food companies and SSB brands to provide additional healthcare resources are seen by our interviewee as a major part of the problem, especially when such companies provide services to people who otherwise cannot afford them.

#### Nutritional colonialism

One way to make sense of this takeover of public spaces and public services by private food conglomerates is the concept of nutritional colonialism [14]. Nutritional colonialism explains contemporary and continuing colonial dynamics which results in the suffering of people in post-colonial contexts from the tactics that transnational food cooperations use to increase their profits. The concept is particularly useful for understanding the context of neo-liberal Jamaica, created as a consequence of Structural Adjustment Programmes that forced the government to deregulate and reduce public spending and therefore cut public services including in health and education [40]. The process of decentralisation and increased use of private sector and third sector health providers led to a decline in funding for primary and secondary health care and reduced percentage in government spending [40]. This means the private sector can more readily fill gaps left behind by the state. As one of our interviewees (a nutrition researcher) stated,

*In all settings where, yes, education is very under-budgeted and health is under-budgeted, it’s hard for governments to say no, we will not take your money. But I think what needs to happen is, that they just need to put in some kind of policy or restrictions, you know, that prevent this. If you want to give money, then it should be provided without that [free advertising].*

-*Nutrition researcher*

The state here is described as an actor with little economic leeway and which is open and vulnerable to corporate practices that harm health [15, 41]. Burnett suggests that while food conglomerates normalise Euro-American foods and consumption habits, and undermine local, healthy, seasonal foods, these dynamics are deeply historical and have had disproportionate effects on colonised peoples [14]. She argues that conglomerate food corporations dominate domestic food systems, unfettered and unregulated in post-colonial contexts. However, in Jamaica, locally owned fast-food companies and sweetened beverage brands also contribute to the domestic sugar economy and environment, and benefit from reduced trade restrictions [32]. Thus cultural changes in consumption habits and desires in post-colonial context can be thought of as another legacy of nutritional colonialism.

### Regulation

#### Resisting taxation and regulation

*We actually got very far; you know [in introducing a sugar tax in Jamaica]. We were this close, this close, believe it. But, you know, the industry is powerful, and they have their tentacles within the government in terms of the beverage industry, and they fought us.*

-*Leader of civil society health organisation*

As the Jamaican government has partnered with corporations, it has also resisted action towards greater regulation or taxation of their practices and public health impacts. A case in point is a sugar tax or levy. Whilst other Latin American countries such as Mexico and Caribbean countries such as Barbados successfully introduced a tax or levy on SSBs, with revenue raised often earmarked for use on NCD policy action or healthcare provision [42–44], policy attempts in Jamaica to do so have ultimately failed. Different views exist on the possible impact of a sweetened beverage levy in Jamaica [45]. For many of our interviewees dedicated to improving the health burden of NCDs, it is a crucial intervention but one that has proven to be politically unviable.

Various explanations have been given for the difficulties experienced with implementing a sugar tax in Jamaica. With a high rate of unemployment and people living in poverty, taxing sweetened beverages has been claimed by some to hurt the poor most and this makes it unpopular [45]. Or as a member of a local health advocacy organisation explained,

*The thing is we’re not going to get anywhere with the tax right now because the government have been campaigning from the beginning on a no tax platform. In fact, there’s a well-known picture we can find within parliament, with them holding up a sign saying ‘no new tax’. This had nothing to do with sugar. it was just their stance on tax…so that is what our problem is, they’re not going to go back on that. And they mean no tax at all, so their whole campaign has been no tax. We just got caught up in that.*

-*Health advocacy campaigner*

Adding to this is a concern amongst some cane farmers that the SSB tax would have a regressive effect and penalise those still trying to grow sugar in Jamaica as a commodity, and thus not just lead to a lowering of consumption. This concern is rooted in the idea that the corporations would not pass the tax on to the consumer but would instead pass it down to the producer. Although this may be an unfounded concern as the refined sugar used in SSBs is mostly imported, there is nonetheless a worry for some farmers we spoke with about anything that appears to be negatively affecting the sugar economy as they already view it as precarious. On the other hand, proponents of a sugar tax in Jamaica argue the income generated from the tax could be invested in much needed health services, especially for diabetes care.

More recently, the effort for industry regulation has shifted towards front of package labelling, a major policy action that civil society actors have been leading throughout the region. However, the Jamaican government has rejected such plans. A nutrition researcher explained to us,

*There has been a lot of pushbacks by industry here. In fact, they’ve [the government] voted against any change [after lobbying]. This update in food labelling standards is not something new. It’s been around from when I started doing nutrition, they’ve been trying to update the food labelling standards, and I know 12 years ago, this was trying to go through, but there was a block then. And for the past three years where we’ve been working on it very intensely, at each stage in the game, they’ve [government] always voted no as a standard.*

-*Nutrition researcher*

Interviewees from the public health field felt there was too much influence of private sector interests in government decision making both due to formal lobbying and exerting power in more informal ways to shape narratives and political thinking.

*What matters to the private sector is the bottom line, they act like they want to be involved to do good, but really they will water down the approach because it hurts their bottom line.*

-*Quote from a nutrition expert at a conference focused on food policy in the Caribbean (2022)*

This is echoed by the 2022 Bertelsmann Stiftung’s Transformation Index report for Jamaica which expressed concern “about the dominance of powerful private sector interests and their ability to influence public policy decisions” (46, p.3). Although the lobbying power of the food sector is strong in many countries, in some countries, civil society has been able to successfully advocate for governments to impose taxes on products high in sugar content, transfats, as well as other products such as alchohol and confectionary [44, 47].

#### Resisting corporate influence

Civil society organisations are significant actors for change in Jamaica, leading major advocacy and community engagement work to reduce the health burden of NCDs. Their campaigns focus on educating the public, but in doing so directly challenge the influence of and narratives otherwise dominated by the private sector. In 2018, a small health NGO experienced a direct backlash by the largest distributor of SSBs in the Caribbean [48]. A public campaign by the NGO included a flavoured water drink and highlighted the many teaspoons of sugar it contains. The inclusion of a specific named product in the health campaign was an error; the NGO did not want specific brands to be named - not due to fears of legal suits but because so many branded drinks have a high sugar content that to tarnish one would fail to address the problem that lies with them all. However, the inclusion of a named product drew such public attention that the company took the charity to court, only to withdraw at the final moment. Threatening the reputation of an SSB by revealing its true sugar content justified initiation of a costly legal challenge by the company [49].

In a review of mass media campaigns, authors showed that such campaigns can produce positive changes or prevent negative changes in health-related behaviours across large populations if they run alongside provisioning of required services and products, community-based programmes, and policies that support behaviour change [50]. However, they also propose that these require investment in longer and better-funded campaigns to achieve adequate population exposure to media messages across. Amongst our interviewees, many shared a feeling that public health campaigns had been effective in changing their own consumption habits but were unsure they had a broad enough reach. One of our interviewees who works in health advocacy reflected on the unequal power dynamics between their counter-campaigns and the continuous efforts of SSB brands advertising.

*Maybe it’s a kind of guerrilla marketing that we have to do, but I just don’t know how to do that effectively. And I’m not seeing that... you know, this was a one-off thing. This guy just did that as a one-off [Drink Yourself Sick campaign], but there’s no structured, effective response to the marketing. And so there’s a view that we will never be able to compete in marketing, so we should go to taxation and regulations. That’s what countries can do.*

-*Health and nutrition campaigner*

Another explained that while health campaigns funded by NGOs or charitable organisations lead the way in advocacy and awareness raising, their campaigns have a finite ‘shelf-life’ when they are dependent on external funding (such as Bloomberg). In this excerpt, they direct a question to our Jamaican interviewer, asking the last time she saw this health advert in Jamaica.

*I think the Bloomberg campaign has had some effect, you know, but I haven’t seen a lot of that advertising recently. It was very prominent, right? Can you think of the last time you have seen that ‘are you Drinking Yourself Sick ad’? It was like a campaign for six months or a year and then it stopped. So, you know, [transnational soft drinks company] never stops. So if you stop, [their commercial advertisement] is going to just overwhelm you again.*

-*Health and nutrition campaigner*

## Discussion

### Summary of findings

In this study, we used an online ethnographic approach and situated Jamaica’s contemporary NCD health crisis within a long and recent historical context. We asked, how can a historical perspective of the determinants of high level SSB consumption in Jamaica contribute to an enhanced understanding of the contemporary context of public health policies and actions aimed at reducing their intake?

We discussed in what way the long history of Jamaica’s sugar plantation economy privileged sugar as a cash crop over domestic food production. We also set out how more recent historical developments such as Structural Adjustment Programmes led to trade deregulation and opened up the Jamaican food and beverage market to transnational companies while also making it easier for these companies to advertise their products. Any government action in terms of taxation through a sugar levy or regulation such as front of package labelling has experienced strong push back by the food and beverage industry, shaped through the long-standing power imbalances between the state and transnationals over time. Finally, we described in what way civil society efforts are challenged by these barriers, and how these are overshadowed by the advertising budget of transnational corporations. Nonetheless, civil society organisations are critical actors to advance the need for structural changes and shift power dynamics.

### Historicising commercial determinants of health

Much of the explanation of SSB consumption in Jamaica and elsewhere can be found within the commercial dynamics that are a main focus of the *commercial determinants of health* field [15] that identifies and analyses “strategies and approaches used by the private sector to promote products and choices that are detrimental to health” [41, p. e895]. These may include excessive marketing towards children and marketing in general [51], lobbying against state regulation [52] and global dynamics of trade [15]. ‘Big Food’ and their soft drink production and marketing have become an area of critical interest to health sciences much like ‘Big Tobacco’ [53]. Yet rarely have studies into ‘Big Food’ adopted a historically grounded approach [53] as we offer here.

We situated our analysis in critical literature around colonial legacies in current food, nutrition and health contexts including work that grounds contemporary ecological analysis in historical explanations [54]. Within the *commercial determinants of health* field, temporal perspectives tend to centre on histories of industry ‘playbooks’, that is histories of how industry actors, most famously of the tobacco industry, have developed deliberate strategies over time to raise doubt about scientific consensus, lobby decision makers and fund counter-narratives, with the aim to limit government action [55]. Early attempts of such harmful strategies have then been adopted and adapted by other industries such as ‘Big Food’ and fossil fuel companies to discredit action against climate change [55]. We would encourage the field to take an even broader historical perspective, and suggest to analyse the global market forces that historically shape power relations in chosen areas of commercial determinants of health research [15].

In our study, we suggest understanding the link of the island’s colonial legacies of sugar-cane production and more recent disruptive economic policies of structural adjustment, which still shape modern-day marketing of branded drinks and barriers to regulation. This is not to deny the complex details that make up such dynamics but to contend a grounded, ethnographic and historical lens can show how such dynamics are configured. For instance, the long existing consumption of sugar in home-made drinks in Jamaica is relevant to the contemporary persuasion of Jamaicans to drink SSBs, and the long-established sugar economy is also part of this persuasion. Hence to understand contemporary advertising and branding of SSBs, it is also necessary to understand the particularities of the local historical and cultural context [56].

Burnett argues that the global obesity epidemic in post-colonial contexts is due to *nutritional colonialism* as transnational conglomerates profit directly from selling highly processed and affordable products to urban working populations and price out local food cultures while changes in lifestyle (as rural populations become absorbed into urban waged labour) limit time to acquire nutritious foods and cook them at home [14]. This seems aptly applied to the case of Jamaica, where structural dynamics have produced the conditions for the commercial sector to have such an influence. As we have shown, trade liberalisation (as deemed necessary for ‘economic growth’) and reduction in public spending limits government action on the proliferation of mass advertisement while making the taxing of harmful products near impossible. Further attempts to increase consumer awareness, such as front of package labelling equally suffer from lobbying from the private sector making any attempt to protect the consumer from *nutritional colonialism* deeply challenging.

Some scholars such as Hatch et al. (57, p. 596) have taken the historical context of colonialism further by acknowledging the racialised aspects of these economies whereby “sugar is guided into Black bodies”, thus bringing historical dynamics of racialisation and enslavement into the contemporary economic and social moment. As they suggest (16. p. 595), “sugar ecologies (are) the product of an unequal and racist system of food production and distribution. We trace the flow of sugar from its sources in the postcolony and racial capitalism, into sociocultural systems we call sugar ecologies, the social systems that drive global sugar production, industrial processing, patterns of distribution and consumption.” While we focused on nutritional colonialism to speak to the specifics of Jamaica’s history, the concept of sugar ecologies adds a potentially vital layer of understanding to this and we suggest such perspectives are given due consideration.

### Limitations

Some limitations to our research must be acknowledged. This has been a small pilot project undertaken under severe logistical restrictions of the covid-19 pandemic. We could not undertake an in-depth ethnographic study, and although this opened up new opportunities, particularly in the increasing availability of online webinars and discussion fora around this pressing public health challenge in the country and region, a more immersive anthropological study of local food/drink practices was not possible. Methodologically, transdisciplinary research is challenging as each discipline has its own epistemological and ontological perspectives. Here we have sought to balance between our different knowledge frameworks and research methods and an ethnographic approach has enabled the required flexibility to do so.

## Conclusion

Research on the marketing practices of unhealthy products and corporate strategies for impeding policy change must be contextualised within globalisation and free trade policies [58] and historicised as part of ongoing colonial dynamics [14, 57, 59]. Our interviewees placed special emphasis on the trade and importation of sugar and unhealthy products into Jamaica, and barriers to regulating industry practices. We argue that understanding commercial determinants of health in post-colonial contexts requires situating these contemporary phenomena within long historical, political, and cultural contexts. For instance, much has been written about how the plantation economies of Jamaica and the Caribbean broadly have shaped contemporary political and economic structures, actors and power dynamics [26, 30, 60, 61] but we offer a perspective on how these connect to current health challenges. A global historical perspective is required to explain uneven and unsustainable contemporary food systems [62]. We bring our transdisciplinary specialisms (including anthropology, epidemiology, economics, history, psychology and ecology) together to situate the NCD health crisis in Jamaica within a colonial and post-colonial context of the powerful global and national food industry. As McKee and Stucker argue, corporations’ influence on health lies particularly in the power to shape dominant narratives [63]; the understanding and compelling telling of the historical economic drivers of contemporary public health challenges is thus a vital tool in the public health toolbox for analysis and advocacy.

## Data Availability

The datasets used and analysed during the current study are available from the corresponding author on reasonable request.

## Abbreviations

Covid-19: Corona Virus Disease-2019
IMF: International Monetary Fund
LMIC: Low and middle income country
NCDs: Non-communicable Diseases
NGO: Nongovernmental organisation
SSB: Sugar sweetened beverage
US: United States (of America)
